# The Combined Propranolol/TSST Paradigm – A New Method for Psychoneuroendocrinology

**DOI:** 10.1371/journal.pone.0057567

**Published:** 2013-02-22

**Authors:** Julie Andrews, Jens C. Pruessner

**Affiliations:** 1 Douglas Mental Health University Institute, McGill University, Montreal, Quebec, Canada; 2 McGill Centre for Studies in Aging, Faculty of Medicine, McGill University, Montreal, Quebec, Canada; University of Tasmania, Australia

## Abstract

Upon perception of a stimulus as stressful, the human brain reacts with the activation of the hypothalamus-pituitary-adrenal (HPA) axis and the sympathetic nervous system (SNS), to mobilize energy resources to better cope with the stressor. Since the perception of the stressor is the initial stimulus, a synchronicity between the subjective perception of stress and the physiological stress reactivity should be expected. However, according to a recent meta-analysis, these associations are weak and inconsistent. The goal of the current study was to investigate the interaction between the SNS, HPA and subjective stress perceptions, by introducing an experimental manipulation of this interaction. For this purpose, we combined the SNS inhibitor propranolol with the Trier Social Stress Test, and measured endocrinological and psychological responses to the stressor. Thirty healthy male participants were recruited and randomly assigned to either a propranolol (PROP; n = 15) or placebo (PLC; n = 15) group. All subjects were administered 80 mg of propranolol 60 minutes prior to exposure to psychosocial stress. Salivary cortisol and alpha amylase (sAA), heart rate, blood pressure and subjective stress responses were assessed throughout the study. We observed significantly reduced sAA levels and heart rate increases in the PROP group in response to stress, with no effects of the drug on systolic or diastolic blood pressure changes. In line with previous studies, a significant increase in cortisol was seen in response to the stress exposure. Importantly, the cortisol increase was significantly higher in the PROP group. A typical increase in subjective stress could be seen in both groups, with no significant group differences emerging. Complementing previous work, this study further demonstrates a significant interaction between the HPA and the SNS during acute stress. The HPA activity was found to be elevated in the presence of a suppressed SNS in reactivity to the TSST.

## Introduction

The physiological and endocrine stress response is considered necessary and adaptive, allowing the individual to mobilize energy resources to overcome a threat, however chronic and excessive activation is associated with numerous physiological and psychological disease states like increased risk for diabetes and cardiovascular disease, burnout, depression and chronic fatigue [1]. During a stress response, two main systems in the human body are activated, the hypothalamus-pituitary-adrenal (HPA) axis, and the sympathetic nervous system (SNS). Upon perception of a stimulus as stressful, the hypothalamus secretes corticotrophin-releasing hormone (CRH), which stimulates the release of adrenocorticotropic hormone (ACTH) from the anterior pituitary. This is released into the bloodstream and eventually binds to receptors in the adrenal cortex, which then releases cortisol into the bloodstream [2]. Cortisol is considered one of the main HPA biomarkers in stress research [3].

In addition to the HPA, the SNS is also activated during a stress response. In the brain, this system induces the release of its effectors, epinephrine (E) and norepinephrine (NE), from the locus coeruleus via stimulation from the brainstem [4]–[6]. The SNS can be assessed in various ways, for example, measuring NE and E levels, galvanic skin response, heart rate, blood pressure, or salivary alpha-amylase (sAA) levels [7], [8].

Numerous behavioral paradigms have been used to induce psychosocial stress. To date, the most frequently used laboratory stressor is the Trier Social Stress Test (TSST: Kirschbaum, et al., 1993), a combination of an impromptu speech and mental arithmetic in front of an audience. The TSST elicits a peak cortisol response 20 to 30 minutes after onset, which returns to baseline approximately 60 minutes post activation [3], [9]. In contrast, SNS markers such as sAA, show peak values in close temporal proximity to the stressor, and a rapid return to baseline levels after the cessation of the stressful stimulus [10]–[12].

Recently, increased attention has been paid to the interaction between the two systems. One review has highlighted the anatomical link between the two systems in the central nervous system, with fibers linking CRH releasing neurons in the hypothalamus with NE releasing neurons in the brainstem [13]. Furthermore, NE levels have been shown to affect HPA regulation by stimulating CRH release from the hypothalamus [5], [6]. Although this evidence suggests a significant interplay between the SNS and HPA, the exact mechanism of this relationship under stress remains to be elucidated.

In addition to the physiological and endocrine reaction to stress, there is also the subjective emotional experience of stress [14]. This characteristic of stress is of great importance as the perception of the situation as stressful is the initial trigger of the physiological and endocrinological response to stress. Hence, there should be a strong correlation between the psychological components of stress (the perception of a situation as stressful) and the physiological and endocrine reactivity to stress. However, this relationship is not consistently observed - a recent meta analysis has found this link to be rather weak and inconsistent [14]. The authors observed an association between the emotional experience of stress and cortisol in only 27% of the studies (N = 30), and in only 25% of studies with cardiovascular measures (N = 12) [14]. The authors note the low number of studies and advise a dynamic approach of assessing the psychological aspects of stress, to go beyond a simple pre and post measure methodology.

This discordance between subjective and objective measures is not only observed in stress research, but has also been highlighted in various other disciplines (for example, subjective pain and sleep experiences [15], [16]). Understanding the mismatch between subjective and objective ratings may help better understanding these various states, and eventually lead to improved interventions.

Our group has previously contributed to the literature on the interaction of the physiological and psychological responses to stress. Using a newly developed stress paradigm by combining the dexamethasone suppression test and the TSST, resulting in “The combined Dexamethasone/TSST paradigm,” we could investigate the impact of suppressing the peripheral HPA axis response on the subjective, and the physiological reactivity to stress [17]. In that study, the groups pretreated with dexamethasone the night before testing showed a suppressed cortisol stress response, together with an elevated heart rate throughout the stress protocol compared to the placebo group, suggesting an inverse relationship between the two stress systems [17]. A trend for higher subjective stress responses to stress could also be observed. These results supported the hypothesis of an inverse association between the HPA and SNS, and we thus wanted to follow-up the original study by conducting the reverse manipulation, and suppress the SNS.

Thus, the goal of the current study was to further investigate the interaction between the SNS, the HPA and the subjective stress response by suppressing the SNS using propranolol, tentatively called: “The combined Propranolol/TSST paradigm.” Propranolol is a non-selective β-antagonist (β_1_ & β_2_) commonly used to treat hypertension, anxiety and tremors [18], [19]. β-blockers have been shown to reduce overall cardiac reactivity and salivary alpha-amylase (sAA) [20]–[23]. Previous human studies have shown an increase in cortisol following propranolol administration [20], [21], [24]–[26], however have not included systematic assessments of stress at the endocrine, physiological and behavioral levels in combination with propranolol administration.

We hypothesized that the acute administration of propranolol an hour before the TSST would lead to decreased SNS responses to the stressor, increased HPA responses, and decreased perception of the task as stressful.

## Methods

### Ethics Statement

McGill University's Faculty of Medicine Institutional Review Board approved this protocol. All subjects provided written informed consent prior to entering the study.

### Subjects

Due to the known confounding effects of cycling menstrual hormones on the HPA and SNS, we focused our study solely on men [27]. Accordingly, thirty healthy men between the ages of 18 and 35 (*M* = 23.13 ± 4.64) with normal body mass index (*M* = 22.48 ± 1.59) were recruited through classified ads. All participants were screened over the phone for their medical history and subjects with medical or psychiatric conditions (e.g. diabetes, depression, etc.) or taking medications, known to affect endocrine function, such as glucocorticoids, were excluded. Smokers were also excluded due to the effects of nicotine on the HPA axis [3].

### General Procedure

After an initial screening participants were randomly assigned to one of two conditions: (1) placebo (PLC) (n = 15, mean age  =  23.80 ± 5.20) and (2) propranolol (PROP) (n = 15, mean age  =  22.47 ± 4.09). Each participant was met twice. The first appointment served to explain the study procedures and to complete the consent form. The next appointment then took place at 9h30 or 10h00 a.m. on one of the following weekdays. Upon arrival participants were given either an 80 mg tablet of propranolol or placebo, one hour before the stress session, in a double-blind fashion [28]. During the following sixty minutes participants then completed a battery of questionnaires assessing numerous personality traits known to affect stress reactivity including parental care [29], self-esteem [30], [31], and depressive symptomatology [32], [33]. Participants were then exposed to the TSST [3], rested for an additional 100 minutes, and were then debriefed about the nature of the study.

### Psychological Stress Induction and subjective stress measures

A slightly modified version of the TSST was used in this study. Participants were given the instructions to the TSST in a waiting room adjacent to the testing room. Subjects were told that they would be doing a mock job interview and had 5 minutes to convince a panel of behavioral experts that they were the best candidates for the job. During the instructions the experimenter introduced the panel and informed the subjects that they would be evaluated on verbal and paraverbal skills (tone of voice, pitch, fluency of speech etc.). Participants were also told that their speech would be recorded for a more detailed analysis and that after the 5-minutes interview, the experts would let them know about a second task. Participants then were allowed to prepare the task for ten minutes, and then gave the speech for five minutes, followed by five minutes of mental arithmetic (counting down from a four digit number in steps of thirteen, having to restart upon making an error). A minor modification to the original TSST was introduced by adding an extra 5-minute period to the anticipation phase to allow the participants to fill out additional questionnaires, including the Primary Appraisal and Secondary Appraisal questionnaire [34], a modified version of the Fear of Negative Evaluation Scale [35], and the COPE inventory [36]. Throughout the study protocol in ten-minute intervals, subjects were asked to rate their subjective stress experience by filling out visual analogue scales (VAS). Participants were instructed to mark an “X” on a ten centimeter line asking “How stressed do you feel right now?” labeled from “not at all” (left side) to “extremely” (right side). Ratings were then applied in the form of 2 cm  =  1 unit.

### Physiological, Endocrinological and Psychological Measures

Salivettes (Sarstedt, Quebec City, Quebec, Canada) were used to collect saliva for assessment of cortisol and alpha-amylase throughout the study protocol in ten-minute intervals, beginning twenty minutes before the onset of the TSST, and in parallel to the VAS scales for subjective stress. Cortisol was analyzed with a time-resolved fluorescence immunoassay with proven reliability and validity [37]. Alpha-amylase was analyzed via the enzyme kinetic method described previously [38]. Blood pressure and heart rate were measured by an ambulatory sphygmomanometer (A&D Company, Tokyo, Japan) and finger pulse oximeter (Roxon, Montreal, Canada).

**Figure 1:**
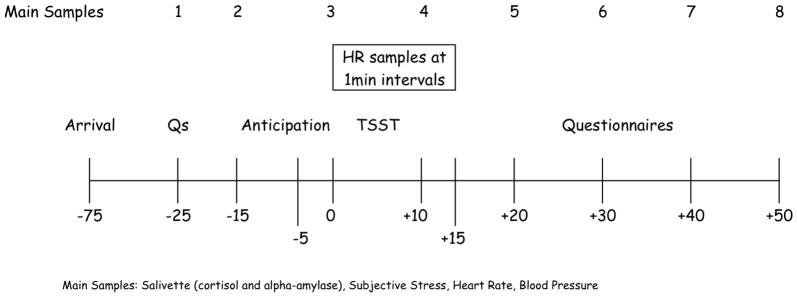
Timeline of the testing procedure.

All variables were measured in ten-minute intervals throughout the protocol except heart rate, which was measured continuously and presented in 1-minute intervals during the TSST and up to five minutes thereafter. Figure 1 depicts the timing of the various samples.

### Statistical Analysis

One-way (group) analysis of variances (ANOVAs) with age, height and weight as dependent variables were conducted to ensure the groups did not differ on these variables.

The interaction and main effects of the experimental conditions on the cortisol levels over time were investigated using a two-factor (time x group) mixed-design ANOVA with the eight cortisol measures as dependent variable. This ANOVA was then repeated for alpha-amylase, subjective stress ratings, heart rate, systolic and diastolic blood pressure, with their eight repeated measurements as the dependent variable. We also performed this analysis with the fifteen one-minute heart rate measures during and after the TSST.

In case of violations of sphericity assumptions, Greenhouse Geisser corrections were performed. Greenhouse Geisser modifies the degrees of freedom in the ANOVA and consequently the significance (p) value. In case of significant main and/or interaction effects, a Tukey's honestly significant difference (HSD) post-hoc test was conducted. All statistical analyses were completed with the SPSS software package16.0.1 on an Apple OS X computer.

## Results

The serial one-way (group) ANOVAs revealed no significant differences between the groups for age and BMI (all *Fs*<1, *ps*>0.05). These results suggest that both groups were comparable on these factors, and that they were unlikely to have contributed to endocrinological and cardiovascular differences between the groups.

### Effects of the Experimental Conditions on Salivary Cortisol

The analysis of salivary cortisol with the two-factor (time x group) *mixed design* ANOVA showed a significant interaction effect of time x group, F_(1.94, 54.26)_ = 4.33, p = .019, a significant main effect of time, F_(1.94, 54.26)_ = 20.355, p<.0001, and a non-significant effect of group, F_(1, 28)_ = 1.769, p>.1.

To further explore the interaction and main effects, we performed a Tukey HSD *post hoc* test. This analysis revealed significantly higher cortisol levels at samples 5 and 6 (post-TSST) in the PROP group compared to the PLC group, both p<.05.

**Figure 2:**
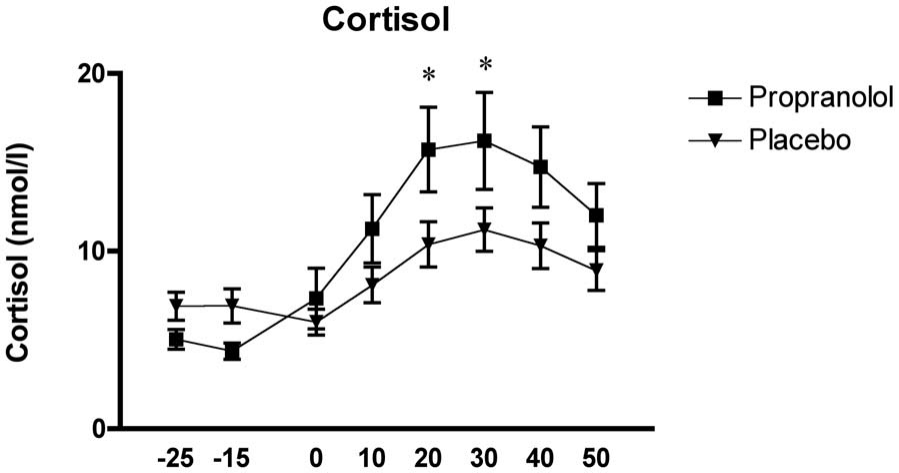
Effects of the TSST on the cortisol response in relation to the two experimental conditions: propranolol (n = 15) and placebo (n = 15).

Furthermore, in both groups, the *post hoc* analysis revealed significant differences between samples 1 through 3 (pre-TSST) and most samples post-TSST and recovery, all p<.01, showing a typical stress reactivity pattern of a cortisol peak 20 to 30 minutes after stress onset (see Figure 2).

### Effects of the Experimental Conditions on Subjective Stress

The two-factor (time x group) mixed-design repeated ANOVA revealed a significant main effect of time, F_(2.84, 79.51)_ = 27.89, p<.0001. All other effects were not significant, all F's<1.74, p's>.1. Thus, in the current sample, we could not show that the suppression of the SNS response to stress through propranolol had a significant effect on the change in subjective stress levels.

**Figure 3:**
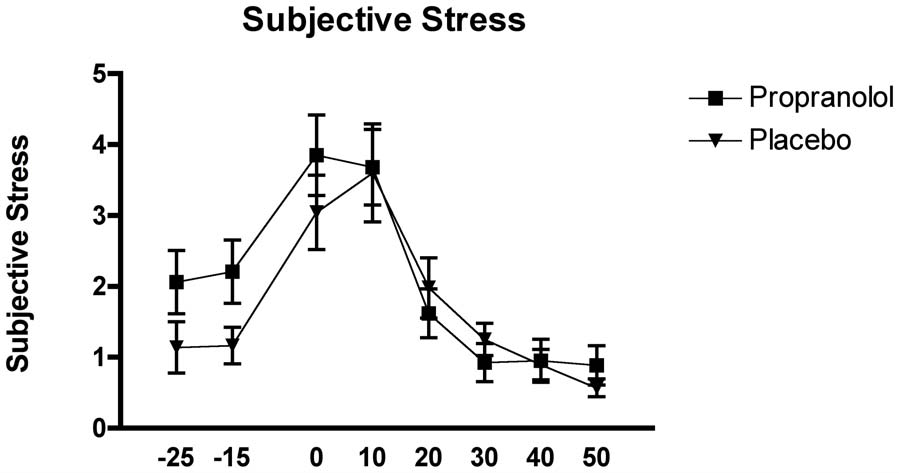
Effects of the TSST on subjective stress ratings in relation to the two experimental conditions: propranolol (n = 15) and placebo (n = 15).

The p*ost-hoc* analysis of the effect of time showed significantly lower samples 1 and 2 (pre-TSST) compared to samples 3 and 4 (pre- and post-TSST), which in turn were significantly higher than samples 5 through 8 (post-TSST), all p's<.0001, overall depicting a response pattern of stress perception peaking immediately pre and post-TSST (see Figure 3).

### Effects of the Experimental Conditions on Salivary Alpha-Amylase

The analysis of the salivary alpha-amylase revealed a significant interaction effect of time by group, F_(3.30, 92.42)_ = 2.774, p = .041, a main effect of time, F_(3.30, 92.42)_ = 8.27p<.0001 and a main effect of group, F_(1, 28)_ = 6.217, p = .019.

The Tukey HSD analysis revealed significant differences at all timepoints between the PROP and PLC groups, with all sAA levels in the PROP group lower than in the PLC group, all p's<0.001.

**Figure 4:**
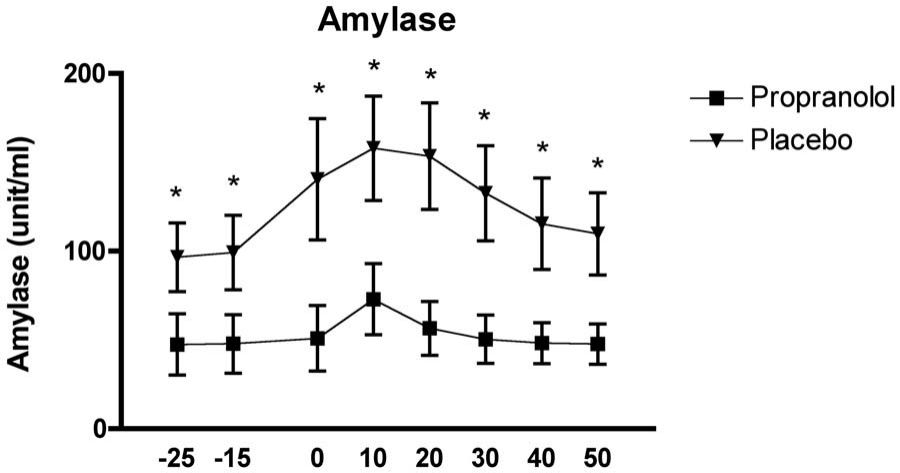
Effects of the TSST on the salivary alpha-amylase response in relation to the two experimental conditions: propranolol (n = 15) and placebo (n = 15).

The *post-hoc* analysis of the effect of time in the PLC group revealed a response pattern with a peak at sample 4 (post-TSST). Samples 1 and 2 (pre-TSST) were shown to be significantly lower than 3, 4 and 5 (pre and post-TSST), and samples 4 and 5 were found to be higher than 7 and 8 (post-TSST), all p<.05. On, the other hand, no significant differences were observed between any of the samples within the PROP group, corroborating the suppressive effects of the pharmacological treatment on sAA (see Figure 4).

### Effects of the Experimental Conditions on Heart Rate

The statistical analysis of the main heart rate measures showed a significant interaction effect of time x group, F_(4.58, 128.34)_ = 2.43, p = .043, and a main effect of time, F_(4.58, 128.34)_ = 16.91, p<.0001, and group, F_(1, 28)_ = 9.97, p = .004.

**Figure 5:**
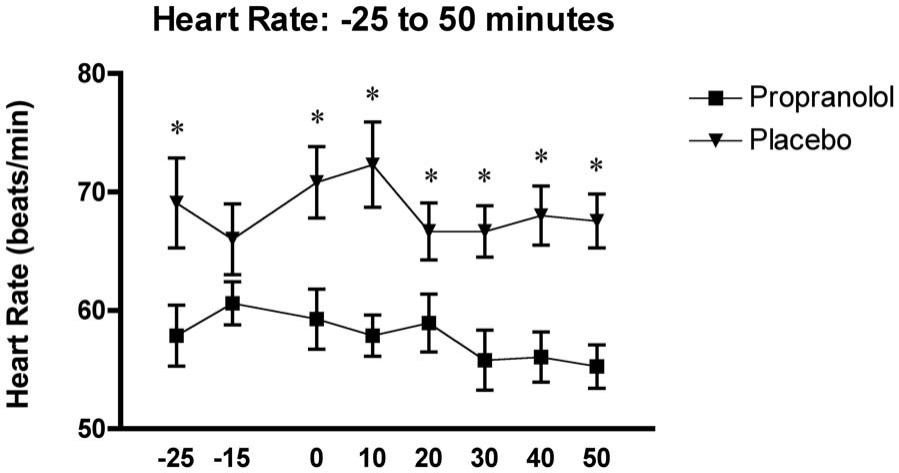
Effects of the TSST on the heart rate in relation to the two experimental conditions: propranolol (n = 15) and placebo (n = 15).

The Tukey HSD *Post-hoc* tests demonstrated that heart rate was found to be higher in the PLC group compared to the PROP group, significantly so at all samples (ps<.001), except for a trend at sample 2, p = .097 (see Figure 5).

Furthermore, the two-factor (time x group) between-design repeated ANOVA with the fifteen 1-minute interval heart rate measures confirmed a significant interaction effect of time x group, F_(3.74, 97.22)_ = 6.02, p<.0001, and a main effect of time, F_(3.74, 97.22)_ = 3.97, p<.0001, and group, F_(1, 28)_ = 23.93, p<.0001.

Similar to the above heart rate results, the *post hoc* analysis indicated that the heart rate measures in the PLC group were found to be significantly higher than in the PROP group, at all samples (p<.01).

**Figure 6:**
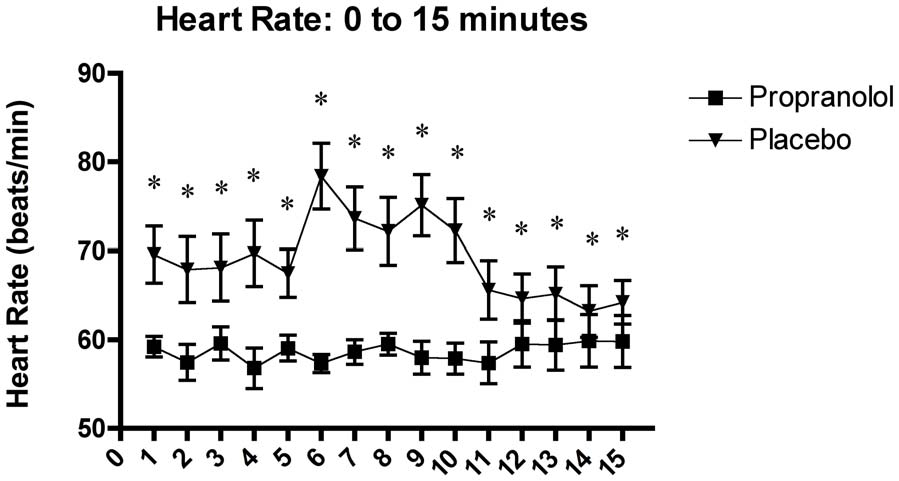
Effects of the TSST on the heart rate in 1-minute intervals during and 5 minutes post-TSST in relation to the two experimental conditions: propranolol (n = 15) and placebo (n = 15).

Moreover, no significant differences were observed among the 1-minute interval heart rate measurements in the PROP group. In contrast, the *post hoc* results within the PLC group heart rate measures showed an increase in heart rate in the middle of the stress session (samples 6 –10) as compared to the recovery period (samples 11 to15). The overall heart rate results confirm the suppressing effect of propranolol on the SNS, where all samples in the PROP group were found to be significantly lower compared to the PLC group (see Figure 6).

### Effects of the Experimental Conditions on Blood Pressure

The ANOVAs with the systolic and diastolic blood pressure levels as dependent variables did not reveal a significant interaction effect of time x group, or main effect of group, for either variable group (all F<4.04, all p>.05). The effect for the systolic blood pressure, F_(1, 28)_ = 4.04, p = .054, approached significance, however. There was a significant time effect for both variables, F_(4.51, 124.62)_ = 14.89, p<.0001, for the systolic blood pressure, and F_(2.53, 70.91)_ = 14.563, p<00001, for the diastolic blood pressure.

**Figure 7:**
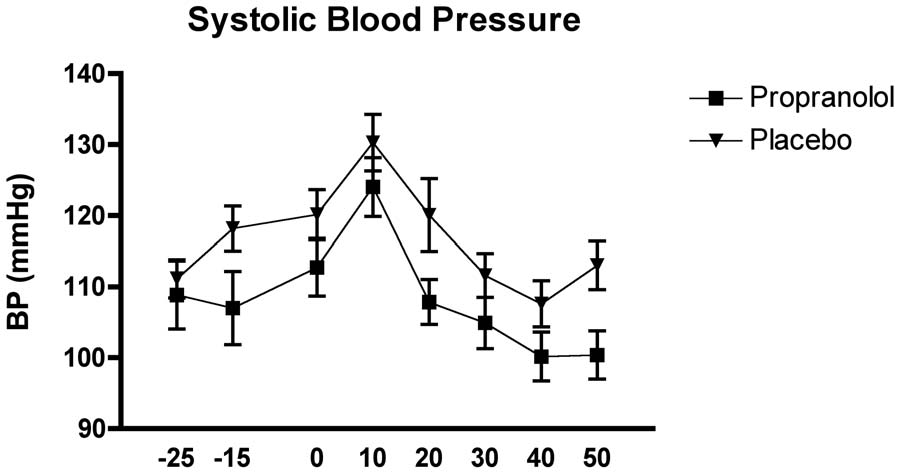
Effects of the TSST on the diastolic blood pressure in relation to the two experimental conditions: propranolol (n = 15) and placebo (n = 15).

**Figure 8:**
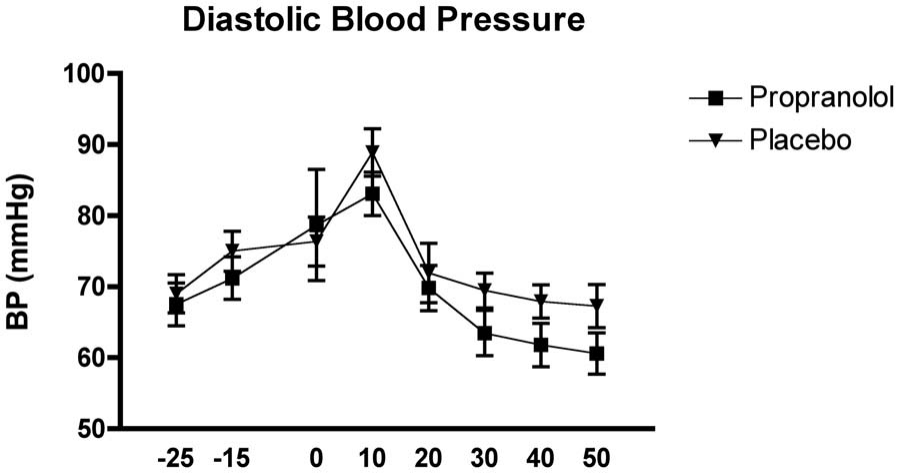
Effects of the TSST on the systolic blood pressure in relation to the two experimental conditions: propranolol (n = 15) and placebo (n = 15).

The Tukey HSD *post hoc* analysis of the time effect for both the systolic and diastolic blood pressure generally showed a similar pattern, overall demonstrating that samples were significantly higher immediately post-TSST compared to baseline and recovery levels, p<0.05. Sample 3 (pre-TSST) was also found to be significantly lower that sample 4, but significantly higher than samples 6 –8, p<0.05 for both variables (see Figure 7 and 8).

## Discussion

In the current study, we aimed to investigate the interaction between the SNS, HPA axis and the subjective emotional stress experience during a psychological stress task. To achieve this goal, we administered a partial SNS inhibitor, propranolol, one hour before conducting a psychosocial stress task, the TSST. This task, the combined Propranolol/TSST paradigm, allowed us to effectively investigate the impact of suppressing the SNS on subjective stress and cortisol responses. Although there are previous studies that have combined propranolol with an acute stress paradigm [20], [21], [24]–[26], these studies focused on the effect of this manipulation on other physiological responses, or various cognitive processes, such as memory, and none have investigated the interaction of all three systems as present in this study.

The significant decrease in heart rate and sAA levels in the PROP group confirms the effectiveness of the pharmacological procedure. However, no significant differences between the groups for the diastolic blood pressure measurements were observed, together with a strong trend for systolic blood pressure. This lack of a clear effect on the blood pressure may be due to the indirect effect of propranolol on the α_1_-adrenoceptor, responsible for vasoconstriction [39]. As propranolol blocks the β_1_ & β_2_ –adrenoceptors, it results in increased synaptic NE and subsequent α_1_-adrenoceptors activation. Accordingly, propranolol is discussed as a direct β-blocker and indirect α_1_ agonist [39]–[43]. Therefore, the combination of these two effects may have mutually antagonized any net effect on blood pressure, explaining the lack of clear group differences on this set of variables.

In line with our finding, other studies similarly reported inconsistent effects on SNS biomarkers changes after propranolol. For instance, Maheu et. al. [24] did not observe significant changes in heart rate and blood pressure after propranolol administration in combination with a TSST paradigm. A potential additional explanation might be that the here used SNS measures were all indirect, thus increasing variability in the target variables, resulting in smaller effect sizes and thus lower chances for significance [17].

The typical cortisol patterns observed in both groups corroborate the effectiveness of the TSST in eliciting a stress response [3]. More importantly, in-line with previous literature, increased cortisol levels were observed in the PROP group [20], [21], [24]–[26]. Different mechanisms have been proposed to explain this effect. First, the SNS could have a bi-regulatory role (excitatory and inhibitory) on the adrenal cortex, where in case of an abnormally elevated cortisol response, the SNS would inhibit the HPA activity [44]. In this case, since propranolol inhibited the SNS, it would have prevented this inhibitory action, explaining the increased cortisol levels in the PROP group. In addition, by preventing NE's binding to the β-adrenoceptors with propranolol, an increased compensatory E production could result, stimulating increased CRH release and subsequent HPA axis activation [44]. The synergistic effect of both mechanisms could explain the rise in cortisol post propranolol administration; nonetheless, further investigation is required to fully comprehend these underlying processes.

The increased cortisol in the propranolol group may have significant implications for the use of propranolol as a treatment for cardiovascular disease and/or social anxiety in the long-term. The chronic effects of excessive cortisol exposure on learning, memory, and cognition are typically considered detrimental, together with adverse effects on neuronal plasticity and dendritic branching. Thus, the short-term benefit of buffered heart-rate response might be offset by the long-term detrimental effects of elevated cortisol exposure. We are not aware of any studies that have investigated this issue, but we think that this is an important topic to consider for future research.The observed subjective stress response findings are is in line with previous studies, where peak subjective stress levels were observed during the TSST, and declined quickly thereafter [45]. Additionally, in the current study subjective stress levels did not appear to be closely related to either HPA, or SNS activity. This was not in line with our expectations, and we thus have to reject the hypothesis that SNS suppression would be associated with lower levels of subjective stress. While the PROP group failed to show an increase in heart rate in response to the stress, this group did show a normal increase in subjective stress levels. Further, in line with previous studies, the magnitude of the cortisol response was found unrelated to subjective stress levels, suggesting a relative independence of subjective stress levels from the activity of the HPA axis. It is of course conceivable that other markers of HPA axis activity, like CRH, would show a closer correlation with the subjective stress levels. The fact that we looked at the most downstream marker of HPA axis activity (cortisol), which also is in large temporal distance to the onset of stress, might have further masked this relationship. Of note, methodological approaches like cross-correlations have been suggested to bridge the temporal gap, and make the cortisol/subjective stress relationship more visible [46].

The relationship between perception of stress and its associated physiological response has been dealt with on an empirical as well as theoretical level. The two-factor theory of emotion, for example, proposes that the perception of the bodily arousal contributes to the emotional. In the context of stress, a decreased arousal (through partial SNS suppression) should thus have led to a decreased subjective stress experience. Our current findings can not support this theory. First, independent of SNS suppression, the appraisal of the stressor as threatening led to a subjective stress response. Second, the levels of subjective stress were even nominally higher in the propranolol group at the onset of the experiment (although this difference was not significant), suggesting if anything an effect in the opposite direction. It could be speculated that this might have been due to a central effect of CRH, since the HPA axis activity at the time of the stressor onset is limited to CRH, which was likely increased at this time already. However, CRH was not measured in the current study, thus this has to remain speculative at this point. Since CRH measures can only be obtained through lumbar puncture, which is rarely feasible within the limitations of a laboratory stress task, an experimental design where both systems are suppressed in the presence of a psychological stress would allow to test this hypothesis instead.

One of the limitations of the current study is that we focused solely on men. Due to the known regulatory effects of hormones involved in the menstrual cycle on the HPA and SNS in women, we decided to first establish the main effects in a male population sample. As a consequence, our conclusions are limited to males only. Future studies should include women in various phases of the menstrual cycle, and women using birth control. Another limitation is the testing time - cortisol stress reactivity is best tested in the afternoon, when levels are lower and allow for a greater cortisol increase over baseline. In the current protocol however, the morning testing time was chosen to be comparable to other ongoing studies in the laboratory. One of these studies employed the dexamethasone suppression test, where dexamethasone was administered at night and the subsequent stress testing had to be done in the morning while the system is still suppressed. Therefore, to allow for later comparison between the different drug regimens (dexamethasone, propranolol and placebo), time of testing was kept identical across all groups.

In conclusion, the combined Propranolol/TSST paradigm allows to investigate the interaction between the SNS, HPA and subjective experiences during acute stress. The results suggest an inverse relationship between the SNS and HPA, where the suppression of the SNS leads to an increase of activity of the HPA. Future studies should explore the combination of using both propranolol and dexamethasone, together with the TSST to further investigate the underlying mechanisms among these stress systems. Finally, the Propranolol/TSST paradigm could be especially useful when aiming to detect possible dysregulations of the HPA axis in response to psychological stress, since the use of the Propranolol amplifies the hpa axis response to psychosocial stress.
